# Ischemic stroke as an initial performance of polycythemia vera in young adults: A case report and literature review

**DOI:** 10.1097/MD.0000000000036953

**Published:** 2024-02-16

**Authors:** Shuo Hui, Jingru Zhao, Tiantian Huo, Lipeng Dong, Yanzhao Xie, Xinyao Wang, Manli Zhang

**Affiliations:** a Department of Graduate School, Hebei Medical University, Shijiazhuang, Hebei, China; b Department of Neurology, Hebei General Hospital, Shijiazhuang, Hebei, China; c Biology Post-doctoral Research Stations, Hebei Normal University, Shijiazhuang, Hebei, China; d Department of Graduate School, North China University of Science and Technology, Tangshan, Hebei, China; e Department of Critical Care Medicine, The Second Hospital of Hebei Medical University, Shijiazhuang, Hebei, China.

**Keywords:** diagnosis and treatment, ischemic stroke, JAK2/V617F mutation, JAK-STAT pathway, polycythemia vera

## Abstract

**Introduction:**

As the second leading cause of death and disability worldwide, stroke is mainly caused by atherosclerosis and cardiac embolism, particularly in older individuals. Nevertheless, in young and otherwise healthy individuals, the causes of stroke can be more diverse and may include conditions such as patent foramen ovale, vasculitis, coagulopathies, genetic factors, or other undetermined causes. Although these other causes of stroke account for a relatively small proportion compared to ischemic stroke, they are becoming increasingly common in clinical practice and deserve attention. Here, we present a rare female patient with polycythemia vera (PV) who was admitted to the hospital as a stroke patient without any previous medical history.

**Patient concerns:**

A 40-year-old young woman felt sudden dizziness and slow response. After 4 days of being admitted, she developed blurry vision on the right.

**Diagnoses:**

Cranial magnetic resonance imaging revealed aberrant signals in the left temporal and parietal lobe, as well as multiple small focal signal abnormalities were observed in the left frontal lobe. Magnetic resonance angiography revealed partial stenosis of the left internal carotid artery. The patient’s blood routine examination revealed a significant elevation in complete blood counts, particularly the increase in red blood cells, as well as prolonged clotting time. An abdominal ultrasound and abdomen computed tomography showed splenomegaly. The outcome of the genetic testing was positive for the Janus kinase JAK2 exon V617F mutation (JAK2/V617F). The patient was diagnosed with PV-related stroke.

**Interventions:**

The patient was treated with phlebotomy, cytoreductive therapy, and low-dose aspirin antiplatelet therapy and was regularly followed up in hematology and neurology clinics after discharge.

**Outcomes:**

The patient’s red blood cell, leukocyte, and thrombocyte counts had fully normalized, with her hemoglobin level measuring at 146 g/L and hematocrit value at 43%. Furthermore, there had been a significant improvement in neurological symptoms.

**Lessons:**

PV, a rare hematological disorder, can present with ischemic stroke as the initial performance, and the diagnosis mainly relies on routine blood tests, bone marrow biopsies, and genetic test. Therefore, clinicians should pay attention to PV, a low-prevalence disease, when encountering stroke in youth.

## 1. Introduction

As the second leading cause of death and disability worldwide, stroke is mainly caused by atherosclerosis and cardiac embolism,^[1]^ particularly in older individuals. Nevertheless, in young and otherwise healthy individuals, the causes of stroke can be more diverse and may include conditions such as patent foramen ovale, vasculitis, coagulopathies, genetic factors, or other undetermined causes. Although these other causes of stroke account for a relatively small proportion compared with ischemic stroke, they are becoming increasingly common in clinical practice and deserve attention. Ischemic stroke associated with hematological disorders is relatively rare, especially among young stroke patients. Polycythemia vera (PV) is a relatively rare chronic myeloproliferative blood disorder, whose diagnosis mainly relies on conducting routine blood tests, bone marrow biopsies, and genetic detection. Its initial presentation may be ischemic stroke, which requiring more attention. The primary risk factors for stroke in PV patients are age, JAK2 exon V617F mutation (JAK2/V617F), as well as previous history of thrombosis, and early diagnosis can improve the management and clinical outcomes of patients. Presented herein is a rare case of a young female patient with PV who was admitted to the hospital with ischemic stroke; however, there was no prior history of atherosclerosis.

## 2. Case presentation

A 40-year-old young woman went to the emergency department on May 26, 2022, after suffering sudden dizziness and delayed response for 1 day. She felt dizziness, accompanied with slow in reaction initially. Her husband complained of his wife incapable of finding the way home, totally understanding what he said and remembering what she just said. The symptoms progressed without nausea, vomiting, diplopia, blurry vision, or swallowing difficulty. There were also no symptoms of sensory dysfunction, focal weakness, or headache, and no prior history of consciousness or seizures. Neurologic diseases were not found in her family history. After being admitted to the hospital on May 30, the patient developed blurry vision on the right side. A neurological examination demonstrated short-term memory and visuospatial skills deterioration, incomplete sensory aphasia, and right hemianopia. Nevertheless, her sensation and limb power were intact, and the Babinski sign was negative on both sides. The ophthalmology consultation confirmed her right hemianopsia.

**Figure 1.**
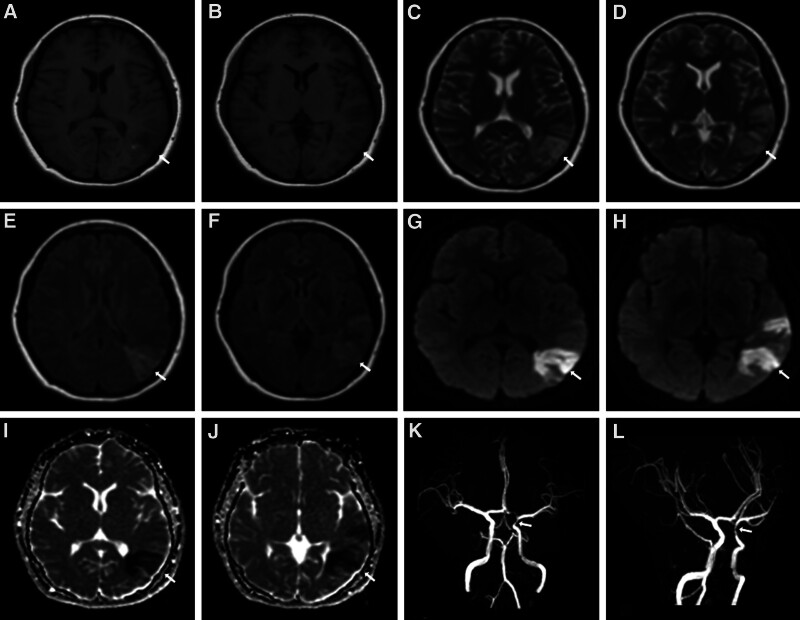
Brain MRI at admission. Axial T1-weighted sequence (A, B), T2-weighted sequence (C, D), FLAIR sequence (E, F), DWI (G, H), and ADC (I, J) sequences were imaged and showed abnormal hyperintensities signals in the left head of temporal, parietal, and occipital lobes. The partial stenosis of the left internal carotid artery in the cerebral MRA (K, L). ADC = apparent diffusion coefficient, DWI = diffusion-weighted imaging, FLAIR = fluid attenuated inversion recovery, MRA = magnetic resonance angiography, MRI = magnetic resonance imaging.

The aberrant signals were presented in the left temporal, parietal, and occipital lobes, with T1-weighted sequence hypointensity, T2-weighted sequence hyperintensity, fluid attenuated inversion recovery sequence hyperintensity, diffusion-weighted imaging hyperintensity, and apparent diffusion coefficient sequence hypointensity at the corresponding position. Magnetic resonance angiography revealed partial stenosis of the left internal carotid artery (Fig. 1A–L). The patient exhibited short-term memory and visuospatial skills deterioration, which was localized to the temporal and parietal cortex. The signs of incomplete sensory aphasia attributed to the left posterior superior temporal gyrus. Moreover, the right hemianopia was ascribed to the optic radiation associated with left occipital lobe. Overall, these symptoms and signs indicated that the issue injuries occurred in the left temporal, parietal, and occipital lobes. Nevertheless, the distribution of these ischemic lesions could not be completely explained by single vessel.

**Figure 2.**
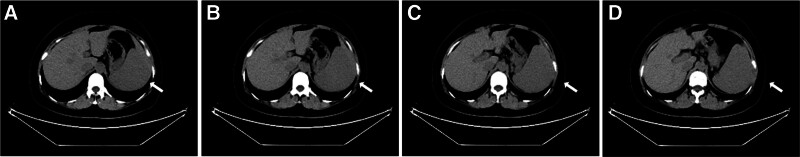
Abdomen CT at admission. An abdominal abdomen CT (A–D) showed splenomegaly. CT = computed tomography.

**Figure 3.**
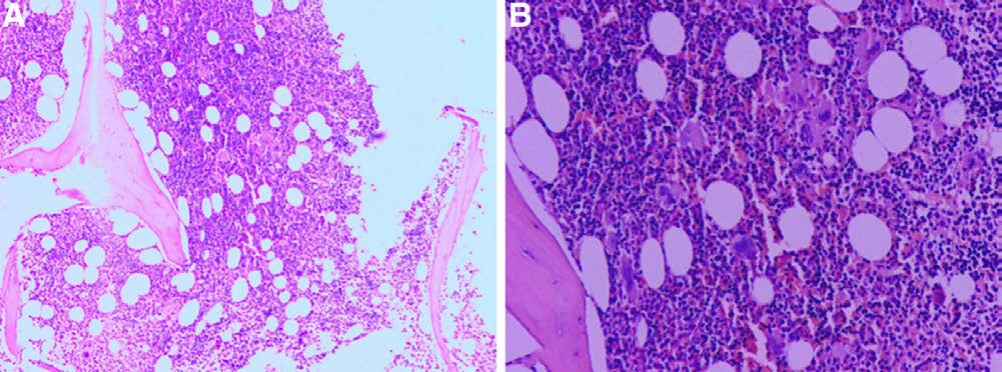
Bone marrow biopsy of the patient. Hyperactive myelodysplasia, hyperplastic megakaryocyte, as well as all stages of granulocytes and erythroblasts were observed. Hematoxylin and eosin staining (A, B).

**Figure 4.**
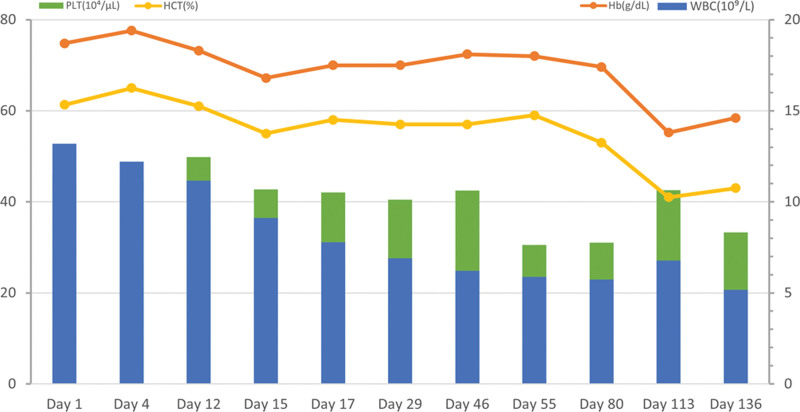
Changes of Hb, PLT, HCT, and WBC from admission to about >4 mo after discharge. Hb = hemoglobin, HCT = hematocrit, PLT = platelet, WBC = white blood cell.

Upon admission, laboratory testing indicated a red blood cell (RBC) count of 7.81 × 10^12^/L, a hemoglobin (Hb) level of 187 g/L, and a hematocrit (HCT) value of 61.3%. White blood cell count was 13.19 × 10^9^/L with 73.60% neutrophils, and platelet (PLT) count was 464 × 10^9^/L. A prothrombin time of 13.3 seconds, an activated partial thromboplastin time of 44.1 seconds, and a thrombin time of 40.8 seconds. The hepatitis and tumor screening, thyroid function, troponin T, N-terminal brain natriuretic peptide, erythrocyte sedimentation rate, and HIV were normal. The lower extremity vascular ultrasound, gynecologic ultrasound, and chest computed tomography (CT) showed no obvious abnormality. The antinuclear antibody test was positive (1:1000), however, the antinuclear antibody profile, antihuman globulin test, antiphospholipid antibody test, antidouble-stranded DNA antibody test, vasculitis screening, immune globulin and addiment test, and lupus anticoagulant test were all negative. During physical examination, the spleen could be palpated 3 cm below the left costal margin. An abdominal ultrasound and abdomen CT both showed splenomegaly (Fig. 2A–D). Thus, based on the increase of complete blood counts, abnormity in the coagulation function, along with the splenomegaly from the abdominal CT scan, a hematological disease such as PV could not be ruled out. Therefore, bone marrow aspiration and genetic testing were conducted in order to clarify diagnosis. The bone marrow biopsy results on May 31 indicated hyperactive myelodysplasia (approximately 80%), reduced granulocyte/erythrocyte ratio, and hyperplastic megakaryocyte (Fig. 3A,B). Genetic testing revealed a quantitative result of 86.1% for the Janus kinase JAK2/V617F mutation. The BCR/ABL fusion gene screening and MPN mutation screening were negative. These results confirmed the diagnosis of PV, and the patient was transferred to the hematology department for further treatment. In consideration of high risk of European LeukemiaNet thrombus score, a combination of phlebotomy, recombinant human interferon alpha cytoreductive therapy was given to the patient. Additionally, a low-dose aspirin regimen (100 mg/d) was also administered as an antiplatelet therapy to mitigate the risk of thrombosis. After treatment, her neurological symptoms such as dizziness, cognitive disorder, and blurred vision improved gradually. After discharge, the Hb level of Hb and HCT significantly decreased to 175 g/L and 58%, respectively. Approximately >4 months after the initial onset of symptoms (October 10), the levels of RBC, white blood cell, and PLT counts fully returned to normal, with Hb level of 146 g/L, and HCT value of 43% (Fig. 4; Table 1). At the same time, her clinical symptoms improved remarkably.

**Table 1:** Laboratory test results of Hb, PLT, HCT, and WBC from admission to about >4 mo after discharge.

	WBC (10^9^/L)	Hb (g/dL)	PLT (10^4^/μL)	HCT (%)
Day 1	13.19	18.70	46.40	61.30
Day 4	12.21	19.40	44.30	65.00
Day 12	11.17	18.30	49.80	61.00
Day 15	9.11	16.80	42.70	55.00
Day 17	7.77	17.50	42.10	58.00
Day 29	6.90	17.50	40.50	57.00
Day 46	6.21	18.10	42.50	57.00
Day 55	5.88	18.00	30.50	59.00
Day 80	5.74	17.40	31.00	53.00
Day 113	6.77	13.80	42.60	41.00
Day 136	5.17	14.60	33.30	43.00

Hb = hemoglobin, HCT = hematocrit, PLT = platelet, WBC = white blood cell.

## 3. Discussion

Ischemic stroke is a serious neurological condition commonly encountered in clinical practice, and its primary etiologies include large-artery atherosclerosis, cardioembolism, small-artery occlusion, other determined etiology, and undetermined etiology. Nevertheless, hematologic disorders cause ischemic stroke relatively rare, accounting for only 1% of all patients who suffer ischemic stroke, and 4% of strokes for young adults.^[2,3]^ PV is a chronic myeloproliferative disorder characterized by uncontrolled production of RBCs, resulting in an increased RBC mass. PV is commonly associated with an increased risk of thrombosis resulting in various complications such as stroke; however, the occurrence of cerebral ischemia in young people with PV is relatively rare.

In a narrative review, in up to 16.2% of PV cases begin with an ischemic stroke, and stroke account for 8.8% of all PV-related deaths.^[4]^ PV patients with stroke are likely to have a multifactorial etiology. Among PV patients, the factors of age, positive JAK2/V617F mutations, and previous thrombosis events are the main risk factors for stroke. The peak incidence of PV occurs between 50 and 70 years old, which is also a period of high-stroke incidence. Furthermore, PV may occur in persons of all age groups, including young adults and children, albeit rarely.^[5]^ The patient in our case was a young woman of 40 years old. The JAK2/V617F mutation is closely associated with PV, and over 95% of patients with PV have this genetic mutation. It produces a protein or enzyme that stimulates the overproduction of blood cells. PV patients are at a heightened risk of thrombotic events, with arterial and venous thromboembolism being the primary contributors to morbidity and mortality in this patient population. The 10-year incidence rate of thrombosis in patients possessing the JAK2 mutation is 21%.^[6]^ Hobbs et al reported that JAK2/V617F mutations might cause endothelial dysfunction to systematic arteries and veins, and promote leukocyte migration and PLT activation, ultimately leading to the formation of emboli that obstruct distal arteries.^[7]^ Moreover, the heightened HCT in PV patients plays a significant role in blood viscosity, which might increase the hemocyte contact to vessel walls, resulting in the formation of localized thrombus and a subsequent increase in the incidence of stroke.^[8]^

The diagnosis of PV primarily relies on blood routine examination, bone marrow biopsy, and genetic detection. World Health Organization 2016 diagnostic laboratory criteria for PV requires that a Hb level of 165 g/L in men and 160 g/L for women or a HCT value of 49% in men and 48% in women and a characteristic JAK2/V617F mutation or JAK2 exon 12 mutation.^[9]^ Nevertheless, in some patients who are eventually diagnosed with PV, the Hb and PLT counts may show a normal-to-borderline increases at the onset of acute stroke, then followed by gradual increase.^[10]^ The patient in our case had no medical history prior to this hospitalization, including a history of atherosclerosis. The patient was admitted to the hospital with ischemic stroke, who was diagnosed with PV based on a Hb level of 187 g/L, HCT of 61.3%, and a positive JAK2/V617F mutation after admission. The bone marrow biopsy revealed hyperactive myelodysplasia, hyperplastic megakaryocyte, and all stages of granulocytes and erythroblasts were observed. The fibrous tissue grade was noted to be 1/3. Therefore, the relationship between cerebral ischemia and PV is worthy of attention, especially in young individuals.

The treatment of acute ischemic stroke in PV is also unique. The primary therapeutic objective in PV is the prevention of thromboembolic events.^[11]^ Antiplatelet therapy can be useful for reducing the risk of thrombosis and its associated complications. The European Collaborative study on PV patients demonstrated the safety and efficacy of low-dose aspirin (100 mg/d) in preventing thrombotic complications, and this study highlighted that patients without contraindications can benefit from this preventive approach.^[12]^ The underlying mechanism might involve the inhibition of aspirin on the interaction between neutrophils and PLTs, thereby reducing the thrombosis risk.^[13]^ A randomized clinical trial focused on the patients with PV demonstrated that HCT levels below 45% could lead to a significant reduction in the incidence of cardiovascular mortality and thrombotic events.^[6]^ Phlebotomy, the removal of excess blood, is highly effective in regulating HCT levels and decreasing blood viscosity and can serve as a primary therapeutic option for PV. It is recommended to consider cytoreductive therapy, such as hydroxyurea or interferon alpha for patients at high risk.^[11]^ Although the precise mechanism remains to be studied, current study shows that these drugs can lead to a reduction in complete blood counts and inflammatory response. Nevertheless, the use of hydroxyurea can exacerbate pancytopenia and increase the secondary risk of neoplasm. Besides hydroxyurea, interferon alpha has been demonstrated to significantly decrease the JAK2 allelic burden.^[14,15]^ Another clinical trial indicated that combining phlebotomy with interferon alpha therapy could result in a noteworthy improvement in HCT levels.^[16]^ In this case, a comprehensive management involving continuous phlebotomy and administration of recombinant human interferon is implemented and achieve a therapeutic target of the HCT below 45.0%.

The Janus kinase-signal transducer and activator of transcription (JAK-STAT) pathway is a crucial signaling pathway involved in various cellular processes, such as cell proliferation, differentiation, apoptosis, and immune regulation. Many cytokines and growth factors, such as hormones, interferon, interleukin, and colony-stimulating factor, utilize the JAK-STAT signaling pathway for transmitting the signals. Inhibition of the JAK-STAT signaling pathway has emerged as a prominent focus in targeted therapy for immune-mediated inflammatory diseases.^[17]^ Dysregulation of the JAK-STAT signaling pathway has been implicated as a significant contributor to various diseases, particularly malignant tumors and autoimmune diseases. Therapeutic interventions targeting the JAK-STAT pathway, particularly JAK inhibitors, have been therapeutically validated in rheumatoid arthritis, ulcerative colitis, ankylosing spondylitis, atopic dermatitis, myelofibrosis, and other diseases.^[18]^ The specific targeted therapies known as JAK2 inhibitors, such as ruxolitinib and phetrotinib, can be employed in case of no response to other treatment or not well-tolerated without considering the mutation of JAK2/V617F. They can significantly reduce blood cell counts, HCT levels, and splenomegaly associated with PV.^[19]^ Ruxolitinib is a reversible JAK1/2 inhibitor with relatively low selectivity. Instead, Phetrotinib is described as a highly specific JAK2 inhibitor, with a stronger inhibitory effect on JAK2 compared with JAK1, JAK3, and TYK2.^[20,21]^ However, the safety profile of phetrotinib is still a subject of debate and ongoing research.^[22]^ The patient’s neurological symptoms, such as dizziness, cognitive impairments, and blurred vision improved gradually after the treatment of phlebotomy, interferon, and low-dose aspirin antiplatelet therapy. The whole blood counts returned to normal about 5 months after the onset, and thus JAK1/2 inhibitor was not used. Nevertheless, the reason for the high level of antinuclear antibodies remains unclear in this patient and might be related to the dysregulation of the JAK-STAT pathway, which play a significant role in immune system.

In summary, clinicians should be aware that PV increases the risk of stroke, and PV-related stroke is a condition with relatively low prevalence but deserves attention, especially in young individuals. If PV-related stroke is strongly suspected, conducting routine blood tests, bone marrow biopsies, and genetic testing can provide a definitive diagnosis. Early diagnosis and management of PV is crucial, as it allows for timely interventions to alleviate symptoms and control disease progression, which is crucial to the patient’s favorable outcome.

## Author contributions

Conceptualization: Shuo Hui.

Data curation: Tiantian Huo.

Formal analysis: Lipeng Dong.

Investigation: Manli Zhang.

Supervision: Jingru Zhao.

Validation: Yanzhao Xie.

Visualization: Xinyao Wang.

Writing - original draft: Shuo Hui.

Writing - review & editing: Shuo Hui, Jingru Zhao.
